# Effect of the Application of the Global Lung Initiative 2012 Spirometry Reference Equation on the Diagnosing and Classifying Degree of Airway Obstruction in Thai Adults Aged 40 to 80 Years Old

**DOI:** 10.3390/medicina55060295

**Published:** 2019-06-21

**Authors:** Warawut Chaiwong, Chaicharn Pothirat, Chalerm Liwsrisakun, Nittaya Phetsuk, Chaiwat Bumroongkit, Athavudh Deesomchok, Theerakorn Theerakittikul, Atikun Limsukon

**Affiliations:** Division of Pulmonary, Critical Care and Allergy, Department of Internal Medicine, Faculty of Medicine, Chiang Mai University, Chiang Mai 50200, Thailand; warawut.chai@cmu.ac.th (W.C.); chalermliw@hotmail.com (C.L.); phetsukn@gmail.com (N.P.); chaiwat.b@cmu.ac.th (C.B.); athavudh.d@cmu.ac.th (A.D.); theerakorn15@yahoo.com (T.T.); atikun.limsukon@gmail.com (A.L.)

**Keywords:** spirometry, equation, pulmonary function, agreement, respiratory

## Abstract

*Background and objective*: Changing to a different spirometry reference equation can result in misinterpretation of spirometric findings. Currently, there is limited data about any discordance between the interpretations of airway obstruction (AO) using the Global Lungs Initiative (GLI) 2012 and the currently employed Thai reference equations (Siriraj) in Thai adults. Therefore, this study aimed to determine differences in diagnosis around AO and classification of the severity of AO using the GLI2012 and Siriraj reference equations in Thai adults. *Materials and Methods*: We analyzed spirometric results from Thai adults aged 40–80 years old (*n =* 2084), which were collected at the Lung Health Center, Maharaj Nakorn Chiang Mai Hospital, Chiang Mai, Thailand between January 2005 and December 2015. The diagnoses concerning the AO were interpreted using the GLI2012 and Siriraj reference equations. The severity of AO in each case was classified into five grades, including mild, moderate, moderately severe, severe, or very severe. McNemar’s test was used to analyze differences in diagnosis of AO and classification of the level of severity. The Kappa statistic was used to determine agreements of diagnosis of AO and classification of severity between the two reference equations. *Results*: There were significant differences in both diagnosis of AO and their classifying severity level between the two reference equations (*p*-value < 0.001). However, the levels of agreement between the two reference equations were moderate to very good in different age and sex groups (Kappa values ranged from 0.62 to 0.78 for the diagnosis of AO and 0.54 to 0.89 for the classification of severity). *Conclusions*: Changing from the Siriraj to the GLI2012 reference equations underestimates the proportion of airway obstruction in Thai adults.

## 1. Introduction

Chronic respiratory diseases (CRDs) are the fourth leading cause of deaths worldwide, in particular in the group of diseases involving airflow obstructions (AO) [1]. In addition to the mortality aspect, AO conditions include chronic obstructive pulmonary disease (COPD) and asthma, both of which have a high socioeconomic impact [2]. The gold standard for diagnosis in cases involving AO is spirometry; specifically, the data calculated from the ratio of forced expiratory volume in the first second (FEV_1_) to forced vital capacity (FVC) less than the 5th percentiles of their relevant predicted values (lower limit of normal, LLN) [3,4]. This FEV_1_/FVC ratio varies between spirometry reference equations due to the differences in the normal subjects being tested from each study. As the interpretation of spirometry results depends on th espirometry reference equations used, these variations will lead to a diversity of opinion [4,5]. Thus, the changing from one spirometry reference equation to another can lead to errors in the interpretation [6,7].

From 2000, the Siriraj reference equations have been established as the main means of interpretation of spirometry results in Thai people [8]. Recently, the European Respiratory Society (ERS) has established the Global Lung Initiative (GLI) 2012 spirometry reference equations, which have been proposed as the first multi-ethnic all age reference equations [9]. The GLI 2012 has also been recommended for use in every country, including Thailand. Previous studies have shown a concordance in the diagnosis associated with AO when using the GLI2012 and the Third National Health and Nutrition Examination Survey (NHANES III) [10,11,12,13], the European Community for Steel and Coal (ECSC) [11], and the Zapletal [14]. However, there is some discordance in the interpretation of AO when changing from the ECSC [10,14,15], and the Stanojevic [10] to the GLI2012. In addition, changing from the NHANES III, the ECSC, and the Stanojevic to the GLI 12 prediction equations will lead to significant changes in the diagnosis of AO in patients with advanced age [10,16,17]. The effect of applying the GLI2012 equations to Thai people is still limited. Therefore, this study aimed to investigate the changes in diagnosis of AO using the GLI2012 compared to the Siriraj reference equations for Thai adults.

## 2. Materials and Methods

### 2.1. Data Collection

We retrospectively collected the pre-bronchodilator spirometry results from Thai adults (aged 40–80 years old) between January 2005 and December 2015. All results that were referred by the pulmonologist and internist for spirometry diagnosis of AO at the Lung Health Center, Maharaj Nakorn Chiang Mai Hospital, Chiang Mai, Thailand were noted. All results were obtained from the subjects with respiratory symptoms (e.g., cough, chest tightness, and dyspnea). Only spirometry results from the first visit of each subject were included in the analysis. There was limited data available for extreme age subjects (aged >80 years old). All spirometry results were collected using a standard spirometer (Vmax series 22, Sensormedics, Yorba Linda, California, USA) and procedures were measured in accordance with the guidelines published by the American Thoracic Society (ATS)/ERS [18]. This study was approved by the Research Ethics Committee of the Faculty of Medicine, Chiang Mai University (Institutional Review Board (IRB), approval number: Med-2559-04324, date of approval: 21 December 2016).

### 2.2. Data Management

Age, sex, height, weight, body mass index (BMI), and body surface area (BSA) were collected from all patients whose spirometry results fitted the criteria [18]. The FVC, FEV_1_, and ratio of FEV_1_/FVC were also collected. The predicted values of FVC and FEV_1_ were calculated from the Siriraj [8] and the GLI2012 (Southeast Asian sub-group) reference equations [9]. The LLN values of the FEV_1_/FVC ratio were also calculated from both reference equations. The disease associated with AO was diagnosed when the ratio of FEV_1_/FVC < LLN [3]. The five severity gradings for AO (mild, moderate, moderately severe, severe, and very severe) were classified based on the percentage of predicted FEV_1_ (%FEV_1_) (≥70%, 60–69%, 50–59%, 35–49%, and <35%, respectively) [3].

### 2.3. Statistical Analysis

A paired sample *t*-test was used for analyzing the differences in the predicted value of FEV_1_, FVC, and LLN values of the ratio of FEV_1_/FVC between the GLI2012 and Siriraj reference equations. Differences in the rate of the diagnosis associated with AO and in the classification of the severity of AO between the two references equations were analyzed using a McNemar’s test. The Kappa (κ) statistic was used for analysis of the agreement on the diagnosis associated with AO between the two reference equations. The Kappa was interpreted as very good, good, moderate, fair, and poor for values of 0.81–1.00, 0.61–0.80, 0.41–0.60, 0.21–0.40, and ≤0.20, respectively [19]. All statistical analyses were performed using STATA version 15 (StataCorp, College Station, TX, USA). The significance level was set at *p* value < 0.05.

## 3. Results

Total acceptable spirometry results from subjects aged ≥40 years old (*n =* 2084) were included for analysis. The sex, age group (40–59 and 60–80), and demographic data of the subjects are shown in Table 1.

### 3.1. Measured Spirometric Values and Predicted Values from GLI2012 and Siriraj References Equations

The mean value of the measured spirometry data from all subjects, predicted values of FEV_1_, FVC, and LLN of the ratio of FEV_1_/FVC from the GLI2012 and Siriraj reference equations are shown in Table 2. The predicted values of FEV_1_ and FVC were significantly higher in the GLI2012 compared to the Siriraj reference equations in both sexes and all age groups (*p*-value < 0.001). The biggest difference in predicted values for FEV_1_ and FVC was found when the results were compared in men aged 60–80 years (220 mL and 200 mL, respectively), but the lowest was found in women aged 40–59 years (60 mL and 110 mL, respectively). The LLN values for the ratio of FEV_1_/FVC were significantly lower in the GLI2012 compared to the Siriraj reference equations in both sexes and all age groups (*p*-value < 0.001).

### 3.2. Detection of Airway Obstruction Using the Two Reference Equations

The rates of the diagnosis associated with AO were significantly higher when using the Siriraj reference equations in all categories, including sex, age groups, and total subjects (*p*-value < 0.001) (Table 3). Changing from the Siriraj to the GLI2012 reference equation resulted in decreasing rates of the diagnosis associated with AO in all categories, including sex, age groups, and total subjects (range from 20.9% to 26.6%).

### 3.3. Severity Classification of Obstructive Airway Using the Two Reference Equations

For the classification of the severity of AO, there were significant differences in the severity of AO when comparing the outcomes of the two reference equations in all categories, including sex, age group, and total number of subjects (*p*-value < 0.001) (Table 3). A higher number of subjects with a mild degree of AO was found when using the Siriraj reference equations in all categories, including sex, age groups, and total subjects (*p*-value < 0.001).

### 3.4. Levels of Agreement for Diagnosis of Airway Obstruction and Classification of Severity Using the Two Reference Equations

This study showed a good level of agreement on diagnosis associated with AO when changing between these two spirometric reference equations (Kappa values ranged from 0.62 to 0.78) (Table 4). The levels of agreement on the classification of severity of AO between the two reference equations were also found to be moderate to very good (Kappa values ranged from 0.54 to 0.89) (Table 4). The highest kappa value for both diagnosis of AO and classification of severity of AO was found in women aged 40–59 years but was lowest in men aged 60–80 years.

## 4. Discussion

This study showed differences in diagnosis associated with AO between the Siriraj and the GLI2012 reference equations in sex, age group (40–59 and 60–80 years old), and total subjects. However, the diagnosis associated with AO when using these two references showed moderate to very good levels of agreement. We suggest that changing from the Siriraj to the GLI2012 reference equations would result in both different diagnosis associated with AO and classification of severity level in Thai adults.

Our study demonstrated that changing from the Siriraj to the GLI2012 reference equations resulted in a change in the rate of the diagnosis associated with AO defined by the ratio of FEV1/FVC < LLN in sex, age group, and total subjects. Changing from the Siriraj to the GLI2012 reference equation resulted in a reduction rate of the diagnosis associated with AO ranging from 20.9% to 26.6% in sex, age group, and total subjects. These results were comparable to previous findings showing that changing from the ECSC and Stanojevic to the GLI2012 equation resulted in a change in the rate of the diagnosis associated with AO [10,14]. Changing from the ECSC to the GLI2012 reference equations caused an increase in the referral rate of AO patients (ranging from 8% to 15.5%) [10,14]. Another study suggested that changing from the Stanojevic to the GLI2012 reference equations caused an increase of 32% in the rate of AO [10]. However, the other studies showed a concordance of the diagnosis associated with AO when changing between the NHANES III and GLI2012 [10,12,16,17] due to the higher LLN value of the FEV1/FVC ratio in the Siriraj reference equation when compared to the GLI 2012 reference equations. Thus, the differences in the rate of diagnosis associated with AO between the two references equations are likely to have occurred when the ratio of FEV1/FVC was close to the LLNs values. For example, in groups with a borderline ratio of FEV1/FVC, there may be a diagnosis associated with AO when using the Siriraj reference equations but not when using the GLI2012 reference equations. This could indicate that the Thai equations may have a higher sensitivity for detecting the diagnosis associated with AO than the GLI2012. The individuals identified as AO from the Thai equations but not from the GLI2012 were classified as discordance of diagnosis of AO and fell into the mild degree of AO category. Culver suggested that spirometry results should be interpreted with caution when the ratio of FEV1/FVC is close to the LLN values [20].

The severity of AO was classified as mild, moderate, moderately severe, severe, and very severe depending on the predicted value of FEV1 from each reference equation [3]. Our results showed a significantly higher predicted value of FEV1 when using the GLI2012 reference equation. The biggest difference in predicted values for FEV1 was found when results were compared in men aged 60–80 years (220 mL) but was the lowest in women aged 40–59 years (60 mL). Therefore, the rates of the severity classification of AO changed when changing from the Siriraj to the GLI2012 reference equation, in particular in the case of a mild degree of impairment. Our results were similar to a previous finding suggesting that the rates of severity classification of AO (COPD and asthma) were changed when changing from the ECSC to the GLI2012 reference equations [13]. However, two other studies showed a concordance of severity classification between the NHANES III and the GLI2012 reference equations [10,16].

The GLI2012 (Southeast Asian sub-group) reference equation was recommended for use for Southeast Asian populations, including Thailand. Indeed, about half of the subjects in Thai spirometry data (*n =* 3954) were included in the establishment of the GLI2012 (Southeast Asian sub-group) reference equations (*n =* 8255) [9]. However, differences in the predicted values of FEV1, FVC, and LLN values of the ratio of FEV1/FVC between the two reference equations were observed. The interpretation of spirometry results is dependent on the predicted values from the reference equations. Thus, the differences in the predicted value of FEV1, FVC, and LLN values of the ratio of FEV1/FVC between the two reference equations may be associated with several factors, including sample size, statistical analysis used, and combined ethnicity. Firstly, differences existed in the sample size used in the two studies. As mentioned before, the GLI2012 reference equations were estimated from the largest sample size (*n =* 8255), whilst the Siriraj reference equations were estimated from 3954 lifetime non-smoker subjects. Differences in sample size used in each study may influence the spirometric predicted value, including the FEV1, FVC, and LLN values of the ratio of FEV1/FVC. Secondly, differences existed in the statistical analysis used between the two studies. The GLI2012 reference equations were calculated using the lambda-mu-sigma (LMS) method whereas the Siriraj reference equations were estimated from regression analyses. Quanjer et al., suggested that the statistics used in the GLI2012 reference equation are the best because they use the biological model of lung function derived from a large population of diverse ages and ethnic populations [9]. Finally, the Siriraj reference equation was derived only from a Thai population, but the GLI2012 reference equations (Southeast Asian sub-group) were derived from four countries, including Taiwan, Southern China, Hong Kong, and Thailand [9]. This combination of several Southeast Asian populations may lead to differences in the spirometric predicted value. Miller suggested that the spirometry predicted values were influenced by ethnicity values [4].

Although, this study showed differences in the diagnosis of AO and classification of the severity level between the Siriraj and the GLI2012 reference equations, the levels of agreement between the two reference equations were moderate to very good in different age and sex groups (Kappa values ranged from 0.62 to 0.78 for the diagnosis of AO and 0.54 to 0.89 for the classification of severity level). Our results were similar to previous findings indicating that there is a good level of agreement in the diagnosis of AO and classification of the severity level between the GLI2012 and the other reference equations, including NHANES III, ECCS, and Stanojevic reference equations (Kappa value ranging from 0.82 to 0.98 for diagnosis of AO [10,16] and 0.95 to 0.97 for classification of severity [16]). However, due to the large percentage of changes in the frequency of the diagnosis associated with AO when changing from the Siriraj to the GLI2012 reference equations (>20% in all categories), the reliability between raters using Kappa values may not reflect the overall outcome of this study. McHugh suggested that the Kappa value should be interpreted with caution when the results of studies may change during clinical practice [21].

The influence of changing from the Thai to the global lung function reference equations is shown in our study. This is a useful result for clinicians and spirometry technicians for increasing the awareness of the outcome of changing the spirometry reference equation used. However, this study does not recommend the best spirometry prediction equation for use in Thai adults but was carried out to inform clinicians that there are important differences in the diagnosis associated with AO and the classification of severity level of AO between the Siriraj and GLI2012 reference equations. In the interpretation of spirometry results, especially for diagnosis associated with AO, the Siriraj and the GLI2012 spirometry reference equations should not be used interchangeably for Thai adult patients.

There are some limitations to this study. The first limitation is that in this study, there are no data for patients of extreme age (>80 years) due to limitations of the available data. Therefore, the results of this study may not be generalizable to Thai subjects aged >80. The second limitation is that in our study, there are no data for subjects with ages between 18 and 25 years old. In this age range, there are significant changes in lung development. Therefore, this age range should be included in future studies. The third limitation is that we did not address the associations between the diagnosis of AO and clinical symptoms of tested subjects. Further studies should investigate the correlations between clinical symptoms and the differences in diagnosis of AO when using different spirometry reference equations. A long-term follow-up needs to focus on the groups in which there are differences in the diagnosis associated with AO when using the two equations. The last limitation is that there are no data regarding smoking history due to limitations of the available data on the electronic files.

## 5. Conclusions

Although there were moderate to very good levels of agreement on the diagnosis associated with AO and classification of the severity level between the Siriraj and the GLI2012 reference equations, changing from the Siriraj to the GLI2012 reference equations underestimates the proportion of airway obstruction in Thai adults. Therefore, the spirometry results should be interpreted with caution when changes in spirometry referencing are used.

## Figures and Tables

**Table 1:** Demographic characteristic data (*n =* 2084).

Variables	Men *n =* 1143	Women *n =* 941	Total *n =* 2084
Number of subjects (%)	1143 (54.8)	941 (45.2)	2084
Age (years)	60.9 ± 10.5	58.9 ± 15.04	60.0 ± 10.5
Age categories, *n* (%)			
40–59 years	538 (47.1)	523 (55.6)	1061 (50.9)
60–80 years	605 (52.9)	418 (44.4)	1023 (49.1)
Height (cm)	161.9 ± 6.5	151.3 ± 5.9	157.1 ± 8.2
Body weight (kg)	58.6 ± 13.1	52.4 ± 11.8	55.8 ± 12.9
BMI (kg/m^2^)	22.3 ± 4.4	22.8 ± 4.7	22.5 ± 4.6
BSA (m^2^)	1.61 ± 0.19	1.48 ± 0.18	1.55 ± 0.20

Data are presented as mean ± SD; BMI, body mass index; BSA, body surface area.

**Table 2:** Measured spirometric values and predicted values from GLI2012 and Siriraj references equations.

Spirometry Variable According to Age Group and Sex	Measured	GLI2012	Siriraj	*p*-Value
FEV_1_ (Liter)				
Men 40–59 (*n =* 538)	2.27 ± 0.73	2.96 ± 0.31	2.79 ± 0.26	<0.001
Men 60–80 (*n =* 605)	1.58 ± 0.61	2.37 ± 0.28	2.15 ± 0.27	<0.001
Women 40–59 (*n =* 523)	1.63 ± 0.53	2.12 ± 0.21	2.06 ± 0.18	<0.001
Women 60–80 (*n =* 418)	1.14 ± 0.43	1.68 ± 0.21	1.59 ± 0.19	<0.001
Total (*n =* 2084)	1.68 ± 0.71	2.32 ± 0.52	2.18 ± 0.47	<0.001
FVC (Liter)				
Men 40–59 (*n =* 538)	3.12 ± 0.82	3.59 ± 0.38	3.48 ± 0.33	<0.001
Men 60–80 (*n =* 605)	2.46 ± 0.67	2.97 ± 0.34	2.77 ± 0.34	<0.001
Women 40–59 (*n =* 523)	2.19 ± 0.59	2.55 ± 0.25	2.44 ± 0.21	<0.001
Women 60–80 (*n =* 418)	1.68 ± 0.48	2.07 ± 0.25	1.95 ± 0.23	<0.001
Total (*n =* 2084)	2.41 ± 0.83	2.85 ± 0.63	2.70 ± 0.61	<0.001
FEV_1_/FVC (%) (LLN)				
Men 40–59 (*n =* 538)	72.30 ± 13.21	72.26 ± 1.39	75.20 ± 1.21	<0.001
Men 60–80 (*n =* 605)	63.51 ± 14.96	67.70 ± 1.66	72.72 ± 1.40	<0.001
Women 40–59 (*n =* 523)	74.22 ± 11.45	73.46 ± 1.09	76.76 ± 0.78	<0.001
Women 60–80 (*n =* 418)	67.06 ± 13.93	69.75 ± 1.48	76.11 ± 0.99	<0.001
Total (*n =* 2084)	69.18 ± 14.17	70.73 ± 2.71	75.06 ± 1.96	<0.001

Data are presented as mean ± SD; *p*-value compared between GLI2012 and Siriraj; GLI, Global Lung Function Initiative; FEV_1_, forced expiratory in first second; FVC, forced vital capacity; LLN, lower limit of normal.

**Table 3:** Detection of airway obstruction and severity classification of obstructive airway using the two reference equations stratified by age and sex.

Detection of AIRWAY Obstruction	GLI2012	Siriraj	*p*-Value
**Age and sex categories**			
Men 40–59 (*n = *538)	204 (37.9)	273 (50.7)	<0.001
Men 60–80 (*n = *605)	337 (55.7)	426 (70.7)	<0.001
Women 40–59 (*n = *523)	204 (39.0)	261 (49.9)	<0.001
Women 60–80 (*n = *418)	215 (51.4)	293 (70.1)	<0.001
Total (*n = *2084)	960 (46.1)	1253 (60.1)	<0.001
**Severity classification of obstructive airway**			
**Age and sex categories**			
**Men 40–59**	*n = *204	*n = *273	<0.001
Mild	77 (37.7)	147 (53.8)	
Moderate	42 (20.6)	44 (16.1)	
Moderately severe	29 (14.3)	23 (8.4)	
Severe	28 (13.7)	33 (12.2)	
Very severe	28 (13.7)	26 (9.5)	
**Men 60–80 **	*n = *337	*n = *426	<0.001
Mild	73 (21.6)	179 (42.0)	
Moderate	61 (18.1)	62 (14.6)	
Moderately severe	48 (14.2)	62 (14.6)	
Severe	99 (29.4)	85 (20.0)	
Very severe	56 (16.7)	38 (8.8)	
**Women 40–59**	*n = *204	*n = *261	<0.001
Mild	79 (38.7)	133 (51.0)	
Moderate	38 (18.6)	36 (13.8)	
Moderately severe	34 (16.7)	40 (15.3)	
Severe	33 (16.2)	35 (13.4)	
Very severe	20 (9.8)	17 (6.5)	
**Women 60–80**	*n = *215	*n = *293	<0.001
Mild	47 (21.9)	109 (37.3)	
Moderate	33 (15.3)	51 (17.3)	
Moderately severe	40 (18.6)	47 (16.0)	
Severe	67 (31.2)	60 (20.5)	
Very severe	28 (13.0)	26 (8.9)	
**Total**	*n = *960	*n = *1253	<0.001
Mild	276 (28.7)	568 (45.3)	
Moderate	174 (18.1)	193 (15.4)	
Moderately severe	151 (15.7)	172 (13.7)	
Severe	227 (23.7)	213 (17.0)	
Very severe	132 (13.8)	107 (8.6)	

GLI, Global Lung Function Initiative.

**Table 4:** Levels of agreement for diagnosis of airway obstruction and classification of severity using the two reference equations.

**Diagnosis AO**	**Kappa (95%CI)**	***p*-Value**
Men 40–59	0.74 (0.69–0.79)	<0.001
Men 60–80	0.69 (0.63–0.75)	<0.001
Women 40–59	0.78 (0.73–0.83)	<0.001
Women 60–80	0.62 (0.55–0.69)	<0.001
Total	0.72 (0.69–0.75)	<0.001
**Classifying Severity of AO**		
Men 40–59	0.79 (0.73–0.85)	<0.001
Men 60–80	0.54 (0.48–0.60)	<0.001
Women 40–59	0.89 (0.84–0.94)	<0.001
Women 60–80	0.79 (0.73–0.85)	<0.001
Total	0.73 (0.70–0.76)	<0.001

AO, airway obstruction; CI, confidence interval.
